# Adherence to Periodic Dilated Eye Examinations and Its Determinants Among Nepalese Patients With Diagnosed Diabetes: A Single-Center Hospital-Based Analysis Using Health Belief Model

**DOI:** 10.1155/2024/3231341

**Published:** 2024-07-30

**Authors:** Barsha Suwal, Rajan Shrestha, Bijay Khatri, Madan Prasad Upadhyay

**Affiliations:** ^1^ Department of Ophthalmology BP Eye Foundation Hospital for Children Eye ENT Rehabilitation Services (CHEERS), Lokanthali, Bhaktapur, Nepal; ^2^ Department of Academics and Research BP Eye Foundation Hospital for Children Eye ENT Rehabilitation Services (CHEERS), Lokanthali, Bhaktapur, Nepal

**Keywords:** adherence, determinants, diabetes, dilated eye examinations, single-center study

## Abstract

**Introduction:** To find the adherence rate to periodic dilated eye examinations (DEEs) and its determinants among patients with diagnosed diabetes.

**Research Design and Methods:** In this cross-sectional study of 165 participants with diagnosed diabetes (Type 1/2) attending a general hospital with a diabetes clinic, we explored perceptions of barriers and facilitators of DEE at the individual level using a framework adapted from the health belief model (HBM). Patients were compared using t tests for continuous data and chi-square tests for categorical data.

**Results:** The rate of adherence to DEE (as defined by DEE *within a year*) was 62.4% (95% confidence interval [CI] = 55.0%–69.8%). The mean age of the patients was 56.81 (±13.29) years. We found that the mean benefit score was significantly higher, and the mean barrier score was significantly lower in those adhering to DEE (*p* < 0.001); but the susceptibility, severity, and self-efficacy scores were not significantly different. Furthermore, those under treatment for diabetes mellitus (DM), those with diabetic retinopathy (DR) in them or their family member, and those with DM duration of 1 year or less were significantly likely to adhere to DEE (*p* < 0.005). Additionally, those who had received advice for eye screening from their physicians were about 25 times more likely to adhere to DEE (95% CI =6.80–92.05) than those who were not advised.

**Conclusion:** A larger proportion of people with diabetes did not adhere to periodic DEE. Benefits and barriers were found to be determinants in this population. Further exploration in a larger population and the use of HBM to increase adherence to periodic DEE can be tested by targeting behavioral counseling along with other traditional approaches.

## 1. Introduction

Diabetes mellitus (DM) is one of the leading causes of avoidable blindness among the working-age adult population [1]. According to a recent meta-analysis, the global prevalence of diabetic retinopathy (DR) among people with diabetes has been reported to be 22.27% (95% confidence interval [CI], 19.73%–25.03%) and is expected to increase to 55.6% to 160.50 million (95% CI, 143.70–178.60 million) in 2045 [2]. The Rapid Assessment of Avoidable Blindness (RAAB) survey conducted in 2010 in Nepal showed that posterior segment diseases were the second major causes of blindness—with DR as a leading contributor [3]. Various studies have reported DR in 19%–47% of diabetic patients in Nepal [4].

Dilated eye examination (DEE) reveals even the first changes in glycemic control and therefore provides opportunities to improve overall systemic care. As a consequence, multiple groups have developed DR guidelines, recommending that diabetic patients should receive regular eye examinations, with the duration between visits based on the severity of retinopathy [5, 6]. Despite recommendations to have regular DEE that allow for early detection and timely treatments that are 90% effective in reducing the likelihood of severe vision loss, it is reported that only half of those diagnosed with diabetes receive an annual eye exam [7, 8]. This rate of adherence in the Western world may not hold true for low-middle-income countries (LMICs), including Nepal. Therefore, this study will bridge this gap of information by finding the rate of adherence to DEE in a single hospital setting in Nepal.

In an effort to increase screening acceptance, public health interventions have targeted traditional barriers to care, such as cost and transportation, with limited success. The health belief model (HBM) is a social cognitive model developed by Rosenstock that is widely used to understand health-related choices [9]. This model considers beliefs and attitudes as proximal determinants of health behavior, including adherence to medical advice [10]. So, this study based on behavioral economics provides a deeper understanding of concepts and tools to understand low screening rates and to promote regular diabetic eye exams.

## 2. Materials and Methods

### 2.1. Study Design and Study Population

The current cross-sectional study was carried out in a general hospital with diabetes clinic in Bhaktapur, Nepal, from September 2021 to April 2022.

A sampling frame was made from the list of general hospitals in Bhaktapur district having diabetes clinic with more than 15 people with diabetes per day, and a hospital was randomly selected through the lottery method. All patients with diagnosed diabetes (Type 1/2), with or without DR, attending the study site during the study period were enrolled. Patients over the age of 18 years, diagnosed as diabetic by physicians, and confirmed by medical records were included. The calculated minimum sample size was 169, calculated using the formulae *n* = *Z*^2^*p* (1 − *p*)/*d*^2^ (*Z* = 1.96 at 95% confidence level, *p* = 0.101 [11], *d* (allowable error) = 0.05, 10% nonresponse rate). All consecutive people with diabetes were included in the study until the required sample size was met.

Ethical clearance was obtained from the Ethics Review Board of the Nepal Health Research Council (protocol registration no. 603/2021 P) prior to the start of the study and was performed according to the principles of the Declaration of Helsinki. Well-informed, written consent was taken from the participants, ensuring their confidentiality and anonymity. The demographic characteristics of the participants such as age, sex, ethnicity, marital status, education level, income per year, and relevant history such as duration of known diabetes and history of DEE in the past 12 months were collected.

### 2.2. Theoretical Framework

**Figure 1:**
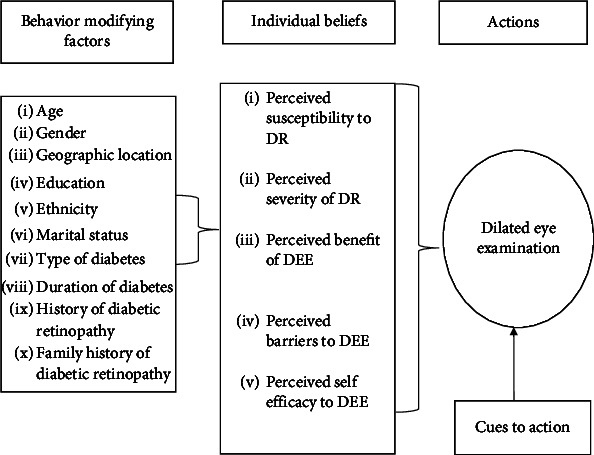
Illustration of determinants of adherence to DEE based on the “Health Belief Model.”

The study was carried out to explore the perceptions of barriers and facilitators of DEE at the individual level using a framework adapted from HBM, which includes modifying factors, individual beliefs, and action/intention for DEE [12]. HBM is depicted in Figure 1. Behavior-modifying factors encompass background characteristics that can influence DEE behavior. Individual beliefs are divided into five constructs: perceived susceptibility, perceived severity, perceived benefit, perceived barriers, and perceived self-efficacy regarding DEE. These constructs were assessed using a 20-item questionnaire on a 5-point Likert scale. Each construct contains four items with a total possible score of 20, except for perceived barriers, which has five items with a total score of 25, and perceived self-efficacy, which has three items with a total score of 15. Responses range from 1 (*strongly disagree*) to 5 (*strongly agree*) for all questions. Examples of questions used were listed below, and detailed questions are included as supplementary attachment.

Examples of questions include the following:

• Perceived susceptibility to DR: “I feel I will get diabetic retinopathy in the future” and “I am more likely than the average diabetic to get diabetic retinopathy.”
• Perceived severity to DR: “The thought of diabetic retinopathy scares me” and “Problems I would experience with diabetic retinopathy would last a long time.”
• -Perceived benefit of DEE: “It is important to have a dilated eye examination annually” and “Regular dilated eye examinations are the best way for diabetic retinopathy to be diagnosed early.”
• Perceived barriers to DEE: “There are many other problems more important than having a dilated eye examination in my life” and “Undergoing a dilated eye examination will make me worry about diagnosing diabetic retinopathy.”
• Perceived self-efficacy for DEE: “It is possible for me to have dilated eye examinations starting next month” and “I am confident that if I want to, I can have dilated eye examinations starting next month.”

### 2.3. Data Collection and Statistical Analysis

The collected data were checked daily for any inconsistencies, then entered into EpiData 3.1, provided with checks to avoid possible data entry errors. The data were then exported to the Social Sciences Statistical Package (SPSS) Version 26.0 for analysis. Patients who returned to the clinic during study period were excluded. Data were collected by a trained enumerator through face-to-face interviews and review of records. Clinical findings were recorded by reviewing outpatient department records and lab reports.

Participants who underwent DEE within the last 12 months were considered to adhere to DEE, for the purpose of the study. Descriptive data on demographic characteristics were generally tabulated and stratified by patients with and without DEE adherence. Comparisons of patients were performed using t tests for continuous data and chi-square tests for categorical data. The adjusted odds ratio for adherence to DEE was calculated through a multivariate logistic regression model, where variables having *p* < 0.1 in the chi-square test were included.

### 2.4. Validity and Reliability of the Tool

The tool was developed according to the study objectives through a workshop with the participation of researchers, public health experts, and retina specialists. An extensive literature review was also performed during tool development. For maintaining the internal consistency of the questions developed on Likert's scale, Cronbach's alpha > 0.7 was taken as acceptable. The reliability score of the HBM construct is illustrated in Table 1.

### 2.5. Pretesting

Pretests were carried out in 10% of the participants (15 patients) in a general hospital with diabetes care in a location other than that used in the study, before the start of the study, for validation. Data were not included in the study.

### 2.6. Patient and Public Participation

Patients or the public were not involved in the design, conduct, or reporting of this research. However, after the interview ended, they received diabetic eye health education.

## 3. Results

Of the total of 170 participants enrolled initially, we could not collect the complete data of five participants; therefore, data from 165 participants were included. Mean age (± SD) of the participants was 56.81 (±13.29) years. Males (58.8%) represented a slightly higher preponderance than females (41.2%). Approximately half of the participants (52.1%) were over 55 years, and only 8.5% of the patients had a known family history of DR. Although most of the participants were literate (91.5%), it was surprising to know that most of them (79.4%) did not know their type of DM. Patients who had known Type 2 DM (16.4%) were 4 times higher than those with Type 1 (4.2%). Mean (±SD) duration of DM (SD) was 7.83 (±6.29) years. Most of the participants had DM for 1–10 years, while more than a quarter (27.3%) had it for more than 10 years. As regard to medical care, more than two-thirds (76.4%) of them were under oral hypoglycemic agents (OHAs), followed by 13.3% who were under OHA and insulin, 7.3% who were not under treatment, and 3% who were under insulin.

Although 78.2% of the patients reported not having DR and a small proportion (4.2% said they had DR in one or both eyes), 17.6% said they were unaware of any retinal changes secondary to DM. The mean (±SD) duration of DR (SD) among those with retinal changes was 1.15 years (±1.33), and 20.6% of those with DR did not report having received DEE advice from their physicians (Table 2).

### 3.1. Adherence to DEE

**Figure 2:**
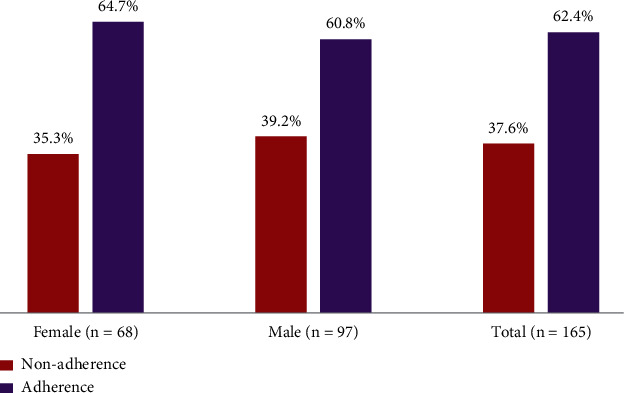
Adherence to dilated eye examination.

Although 79.4% (95% CI = 73.2%–85.6%) reported having DEE at some point in their life, the rate of adherence to DEE was 62.4% (95% CI = 55.0%–69.8%) as shown in Figure 2.

Further analysis was done on the association of the HBM construct and other characteristics with the adhesion of DEE.

### 3.2. Health Beliefs and Adherence to DEE

We found that the mean benefit score was significantly higher and the mean barrier score was significantly lower in those who were adhering to DEE (*p* < 0.001); but the susceptibility, severity, and self-efficacy scores were not significantly different.

The mean benefit score, which is the subjective assessment of an individual's perception of the benefit of DEE, predicts that individuals will go for DEE if they perceive that it is beneficial to them. In our study, we found that patients who considered that they understood the benefits of DEE were more likely to adhere to DEE (*p* < 0.005).

Similarly, the mean barrier score is the subjective assessment of an individual's perceived obstacles for adopting DEE. In our study, we found that patients who considered perceived benefits to outweigh perceived barriers were more likely to be adherent (*p* < 0.005).

However, patients who did/did not consider themselves likely to be affected by DR (susceptibility score), or who thought that even if they had DR, it would not be severe enough (severity score), or patients who thought that they were/were not competent for undergoing DEE (self-efficacy score) were not statistically different in two groups. Similarly, the mean age and duration of DM were not significantly different in two groups. Details on the mean score on HBM constructs according to adherence to DEE are given in Table 3.

### 3.3. Adherence to DEE and Its Other Associated Factors

We found that those who were treated for DM, those with DR in them or in their family member, those who have received advice on eye screening from their physicians, and those with DM duration of 1–10 years were significantly likely to adhere to DEE (*p* < 0.005). However, we did not find any significant association with age, gender, ethnicity, religion, education level, type of DM, visual impairment due to DR, offer of free eye examination, or any information on DR provided through the media. The details of the association with the level of adherence are given in Table 4.

Participants advised for DEE by physician or health workers were 25.02 (95% CI = 6.80–92.05) times more likely to have adherence to DEE than those who were not advised (Table 5).

## 4. Discussion

This study evaluated adherence to DEE and explored the perspective of Nepalese diabetic patients about nonadherence to routine DEE, to improve the general approach to quality diabetic eye care. Of the 165 participants included in the study, the rate of adherence to DEE in a year was 62.4%, implying that about one in three patients missed out on their yearly DEE, similar to the findings in other studies, such as the findings of the National Health and Nutrition Examination Survey (NHANES) from 2005 to 2016, United States, where they reported it as 63.4% [13]. The rate of annual eye exams for DR in our study was higher than in Korea (32.7%) [14] and Kenya [15] where they reported as 13.3%. A study done in the general practitioner's clinic in the urban city of Delhi, India, reported that only 7.4% of people with diabetes had undergone DEE in the previous year [16]. Another community-based representative sample survey of diabetes quality of care from middle- and high-income residents of Delhi, India, in 2005–2006 (DEDICOM) reported a 16.2% DEE adherence rate [17]. This is in contrast to our study, where we reported a higher percentage. It could be because our study was done in a diabetes clinic, where endocrinologists were involved in patient management. The type of physician involved in glycemic control can affect the referral rate to ophthalmologists [18]. A study conducted by Leinung et al. found that adherence to American Diabetes Association guidelines was significantly better when patients were followed by endocrinologists versus primary care providers, where they reported that 90% of patients who were followed by endocrinologists in the study received annual eye examinations versus 50% of those seen in a primary care clinic [18].

However, in a population-based DR survey as part of the RAAB survey in the far-western province of Nepal, it was found that only 27.7% of cases had their eye examined for DR in the last year [19]. This low adherence rate is explained by the fact that this survey was carried out in one of the most underserved areas in the country. This discrepancy compared to our study could be because the participants in our study were mostly urban residents.

Many factors influence adherence to screening guidelines, including access to care, limited understanding of the disease, inadequate referral rates, cost, and transportation [20]. In addition, other pervasive barriers include not perceiving regular eye exams as a priority and not understanding the benefits of having regular eye exams [21].

Based on HBM, we found that behavior-modifying factors such as having DR, those with a family history of DR, those who had received advice on eye screening from their physicians, and those who underwent treatment for DM were more likely to adhere to DEE. In other studies, a longer duration of diabetes was associated with adherence [22], but we found that shorter durations of DM, especially less than a year, were significantly more likely to adhere to DEE. This could mean that the longer the duration of the DM, the lesser the chances of being compliant with follow-up.

Individual beliefs, such as the benefits of having DEE, outweighed the cons, and those with fewer barriers to evaluating the detailed fundus evaluation carried the highest likelihood of adherence. This could mean that we should focus our counseling for DEE by making patients understand the benefits of having regular eye screening and mitigating potential barriers such as distance, cost, having a family or friend to accompany, and availability of service. Many patients did not consider themselves susceptible to developing DR nor thought that DR was serious.

In our study, people who had received a diagnosis of DR were more likely to follow up regularly, similar to the NHANES study [13]. Unsurprisingly, we found that those who received advice for eye screening from their doctors were more likely to follow up, as lack of advice or referral is a well-known major barrier to screening. Likewise, unlike other studies in which a longer duration of diabetes was associated with adherence [22], we found that having DM for a shorter period was more likely to adhere. As the disease became chronic, patients tended to be more negligent of regular eye exams.

Our study has few limitations. Since the patients were asked about the status of DR and the visual impairment, the findings of our study are subjected to information bias including memory bias and social-desirability bias. However, we tried to limit this error by corroborating with the medical records wherever possible. Also, our cohort of patients included mobile patients with medical stability. Therefore, our study could not analyze perceived barriers among immobile patients and patients with medical instability. Additionally, being a single-center study, the results may not be generalized. Despite these limitations, our study determined the adherence rate of DEE in a hospital-based diabetes clinic in Nepal, which was not previously available. The study is unique because it used the HBM to determine factors to determine adherence. The uptake of diabetic screening exams can be increased by addressing the barriers mentioned above, hence closing the loop for those most likely to drop out of regular follow-ups.

## 5. Conclusions

The rate of adherence to DEE was 62.4%. In general, those who thought the benefits of having DEE outweighed the cons and who had less barriers to detail fundus evaluation carried the highest probability of adherence.

On the contrary, the population least likely to have had an eye exam in the past year was those who did not receive advice on eye examination from their physicians; those with long duration of diabetes and those not under DM treatment. Individuals who denied receiving a diagnosis of DR and those who did not have a history of DR in family members also had lower adherence rates. When these issues are addressed, the low uptake of diabetic screening exams can be mitigated.

## Figures and Tables

**Table 1:** Reliability of health belief model constructs.

**Health belief model constructs**	**Number of items**	**Cronbach's alpha**
Susceptibility	4	0.934
Severity	4	0.705
Benefit	4	0.939
Barrier	3	0.729
Self-efficacy	3	0.902

**Table 2:** Participants' characteristics.

**Characteristics**	**Frequency (** **n** **)**	**Percentage (%)**
Gender		
Female	68	41.2
Male	97	58.8
Age group (years)		
26–55	79	47.9
56–85	86	52.1
Educational level		
Illiterate	29	17.6
Can read and write	14	8.5
Primary	19	11.5
Secondary	51	30.9
Higher secondary	12	7.3
Bachelor or above	40	24.2
Marital status		
Married	165	100.0
Type of DM		
Type 1	7	4.2
Type 2	27	16.4
Do not know	131	79.4
Duration of DM (years)		
Up to 1	24	14.5
> 1–10	96	58.2
> 10	45	27.3
Treatment for diabetes		
OHA	126	76.4
Insulin	5	3.0
Both OHA and insulin	22	13.3
No treatment	12	7.3
History of DR		
Yes	7	4.2
No	129	78.2
Do not know	29	17.6
Family history of DR		
Yes	14	8.5
No	124	75.2
Do not know	27	16.4
Health workers' advice for DEE		
No	34	20.6
Yes	131	79.4

**Table 3:** Adherence to DEE based on mean scores of HBM constructs.

**Characteristics**	**Adherence to DEE**		**Nonadherence to DEE**		**p** **value**
	**Mean**	**SD**	**Mean**	**SD**	
Age (years)	56.4	13.4	57.5	13.1	0.596
DM duration (years)	8.0	5.7	7.6	7.2	0.727
Perceived susceptibility	2.6	0.7	2.5	0.7	0.548
Perceived severity	3.2	0.5	3.2	0.5	0.861
Perceived benefit	4.3	0.6	3.8	0.7	< 0.001^a^
Perceived barriers	2.3	0.6	2.6	0.6	< 0.001^a^
Perceived self-efficacy	4.2	0.5	4.1	0.6	0.323

^a^Statistically significant at *p* < 0.001.

**Table 4:** Association of adherence to DEE with other characteristics.

**Characteristics**		**Adherence to DEE**		**Nonadherence to DEE**		**p** **value**
		**Number (** **n** **)**	**Percentage (%)**	**Number (** **n** **)**	**Percentage (%)**	
Gender	Female	44	64.7	24	35.3	0.612
	Male	59	60.8	38	39.2	

Education	Up to secondary level	65	57.5	48	42.5	0.055
	Higher secondary or above	38	73.1	14	26.9	

Type of diabetes	Type 1	5	71.4	2	28.6	0.759
	Type 2	18	66.7	9	33.3	
	Do not know	80	61.1	51	38.9	

Treatment for diabetes	Under treatment	100	65.40	53	34.60	< 0.01^a^
	No treatment	3	25.00	9	75.00	

Diabetic retinopathy	Yes	7	100.0	0	0.0	< 0.001^a^
	No	96	74.4	33	25.6	
	Do not know	0	0.0	29	100.0	

Family history of DR	Yes	11	78.6	3	21.4	< 0.001^a^
	No	90	72.6	34	27.4	
	Do not know	2	7.4	25	92.6	

Visual impairment due to DR	No	98	61.6	61	38.4	0.281
	Yes	5	83.3	1	16.7	

Physicians' advice for DEE	No	4	11.8	30	88.2	< 0.001^a^
	Yes	99	75.6	32	24.4	

Got free eye exam	No	95	60.9	61	39.1	0.092
	Yes	8	88.9	1	11.1	

Got DR information	No	76	59.4	52	40.6	0.133
	Yes	27	73.0	10	27.0	

DM duration (years)	Up to 1	8	33.3	16	66.7	< 0.01^a^
	> 1 to 10	68	70.8	28	29.2	
	> 10	27	60.0	18	40.0	

Age group (years)	26–55	55	69.60	24	30.40	0.067
	56–85	48	55.80	38	44.20	

^a^Statistically significant at *p* < 0.05.

**Table 5:** Adherence to DEE and participant characteristics.

**Characteristics**		**Adjusted odds ratio (95% CI)**	**p** **value**
Education	Up to secondary level	1.84 (0.78–4.35)	0.164
	Higher secondary or above	1	

Treatment for diabetes	Under treatment	1.09 (0.15–7.79)	0.930
	No treatment	1	

DM duration (years)	>= 10	1.14 (0.48–2.69)	0.77
	< 10	1	

Age group (years)	26–55	2.06 (0.91–4.65)	0.083
	56–85	1	

Family history of DR	Yes	1.87 (0.35–9.90)	0.464
	No/do not know	1	

Physicians' advice for DEE	No	25.02 (6.80–92.05)	< 0.001^a^
	Yes	1	

^a^Statistically significant at *p* < 0.001.

## Data Availability

All data relevant to the study are included in the article.

## Nomenclature

CI: Confidence interval
DEE: Dilated eye examination
DM: Diabetes mellitus
DR: Diabetic retinopathy
HBM: Health belief model
LMICs: Low-middle-income countries
NHANES: National Health and Nutrition Examination Survey
OHA: Oral hypoglycemic agents
RAAB: Rapid assessment of avoidable blindness
