# Correction to “Adherence to Periodic Dilated Eye Examinations and Its Determinants Among Nepalese Patients With Diagnosed Diabetes: A Single‐Center Hospital‐Based Analysis Using Health Belief Model”

**DOI:** 10.1155/bmri/9826384

**Published:** 2025-10-31

B. Suwal, R. Shrestha, B. Khatri, M.P. Upadhyay, “Adherence to Periodic Dilated Eye Examinations and Its Determinants Among Nepalese Patients With Diagnosed Diabetes: A Single‐Center Hospital‐Based Analysis Using Health Belief Model,” *BioMed Research International*, 2024, https://doi.org/10.1155/2024/3231341.

In the article, there is an error in Table 5, where the results presented for the Physicians′ advice for DEE are incorrect. The correct Table 5 is shown as follows:

**Table 5:** Adherence to DEE and participant characteristics.

**Characteristics**		**Adjusted odds ratio (95% CI)**	**p** **value**
Education	Up to secondary level	1.84 (0.78–4.35)	0.164
	Higher secondary or above	1	
Treatment for diabetes	Under treatment	1.09 (0.15–7.79)	0.930
	No treatment	1	
DM duration (years)	>= 10	1.14 (0.48–2.69)	0.77
	< 10	1	
Age group (years)	26–55	2.06 (0.91–4.65)	0.083
	56–85	1	
Family history of DR	Yes	1.87 (0.35–9.90)	0.464
	No/do not know	1	
Physicians′ advice for DEE	Yes	25.02 (6.80–92.05)	<0.001^∗^
	No	1	

^∗^Statistically significant at *p* < 0.001.

We apologize for this error.

